# Identification and analysis of key circRNAs in the mouse embryonic ovary provides insight into primordial follicle development

**DOI:** 10.1186/s12864-024-10058-y

**Published:** 2024-02-03

**Authors:** Xiangyan Wang, Yan Zhang, Jianjie Yu, Yabo Ma, Yaxiu Xu, Jiaqi Shi, Zhipeng Qi, Xinfeng Liu

**Affiliations:** 1 Key Laboratory of Ministry of Education for Conservation and Utilization of Special Biological Resources in the Western, Ningxia University, Yinchuan, Ningxia 750021 China; 2 School of Life Sciences, Ningxia University, Yinchuan, Ningxia 750021 China

**Keywords:** circRNA, RNA-seq, Primordial follicle, Ovary, Mice

## Abstract

**Background:**

CircRNAs are a class of noncoding RNAs with tissue- and development-specific expression characteristics. In many mammals, primordial follicle development begins in the embryonic stage. However, the study of circRNAs in primordial follicle development in mice has not been reported.

**Results:**

In this study, ovaries were collected from mouse foetuses at 15.5 days post coitus (dpc) and 17.5 dpc, which are two key stages of primordial follicle development. A total of 4785 circRNAs were obtained by using RNA-seq. Of these, 83 differentially expressed circRNAs were identified. Gene Ontology (GO) and Kyoto Encyclopedia of Genes and Genomes (KEGG) enrichment analyses showed that these differential circRNAs were mainly involved in the regulation of reproductive development. Through qRT-PCR, back-splice sequence detection and enzyme digestion protection experiments, we found that circ-009346, circ-014674, circ-017054 and circ-008296 were indeed circular. Furthermore, circ-009346, circ-014674 and circ-017054 were identified as three key circRNAs by analysing their expression in the ovaries of mice at different developmental stages. The circRNA-miRNA-mRNA interaction network was constructed and validated for target miRNA and mRNA using qRT-PCR. The interacting genes circ-009346, circ-014674, and circ-017054 were subjected to KEGG enrichment analysis. We found that circ-014674 may participate in the assembly and reserve of primordial follicles through oestrogen and the Janus kinase (JAK) signal transducer and activator of transcription (STAT) signalling pathway (JAK-SATA). Circ-009346 and circ-017054 may have similar functions and are involved in the activation and growth of primordial follicles through the mitogen-activated protein kinase (MAPK) and phosphoinositide 3-kinase (PI3K) signalling pathways.

**Conclusions:**

Based on our findings, three circRNAs associated with primordial follicle development were identified, and their potential mechanisms of regulating primordial follicle development were revealed. These findings will help us better understand the molecular mechanism of circRNAs in primordial follicles and provide important references and targets for the development of primordial follicles.

**Supplementary Information:**

The online version contains supplementary material available at 10.1186/s12864-024-10058-y.

## Introduction

The follicle is the basic functional unit of the ovary. According to its developmental stage, the follicle in the ovary can be classified as a primordial follicle, a primary follicle, a secondary follicle, a tertiary follicle or a Graafian follicle [1]. Most female animals establish a pool of primordial follicles around birth, and after sexual maturity, the number of primordial follicles decreases with periodic oestrous [2]. At birth, the number of primordial follicles is approximately 10,000 in mice, 1 million in humans, and millions in other domestic animals. By the time of oestrous, the number of primordial follicles drops to approximately 5,000 in mice, 250,000 in humans, and 40,000 in sheep [3]. In mammals, primordial germ cells (PGCs) migrate to the genital ridge and finally differentiate into germ cells [4]. At 8.5 dpc in mouse embryos, primordial germ cells start to differentiate to a low degree. At 10.5 dpc-13.5 dpc in mouse embryos, syncytium structures are formed, and female germ cells (oocytes) enter meiosis [5]. At 15.5 dpc in mouse embryos, a large number of syncytia are formed, and most oocytes enter meiosis [6]. In mice, the syncytium ruptures to form primordial follicles at 17.5 dpc, and the first batch of primordial follicles is activated during this period [7]. Thus, 15.5 dpc and 17.5 dpc in mouse embryos are critical periods for primordial follicle development.

The formation and activation of primordial follicles determine ovarian function and female fertility. However, the formation of primordial follicles is regulated by a variety of signalling pathways. For example, the MAPK signalling pathway [8], the JNK signalling pathway [9] and the Notch signalling pathway [10] are essential for the formation and maintenance of primordial follicles. In addition, primordial follicle formation is regulated by a variety of proteins and growth factors, such as histone deacetylase 6 (HDAC 6) [11], epidermal growth factor (EGF) [12], and transforming growth factor-β (TGF-β) [13].

At present, research on primordial follicles mainly focuses on specific pathways and proteins, while research on circRNAs is relatively limited. In 1976, circRNA was first proposed as the concept of “covalently closed circular single-stranded RNA” [14]. Later, it was found that circRNAs were composed of introns, exons and noncoding gene intervals [15], and circRNA was identified in tissues and cells of humans [16], mice [17], monkeys [18], *Caenorhabditis elegans* [19], etc., showing characteristics of tissue- and development-specific expression [20]. Most circRNAs were found in the cytoplasm [21], but later, special exon-intron circRNAs were found in the nucleus [22]. Most mammalian primordial follicle development begins in the embryonic stage [7], but the current research on follicle development is focused on the period after birth. It has become clear that the mouse embryonic stage at 15.5 dpc and 17.5 dpc is a critical period for the development of primordial follicles. To date, no circRNAs related to primordial follicle development have been studied using embryonic mouse ovaries. Therefore, in this study, mouse ovaries at these two stages were selected and subjected to circRNA sequencing, combined with bioinformatics analysis and related experiments, to identify the key circRNAs involved in primordial follicle development and reveal their function.

## Results

### Global analysis of sequencing data

**Fig. 1:**
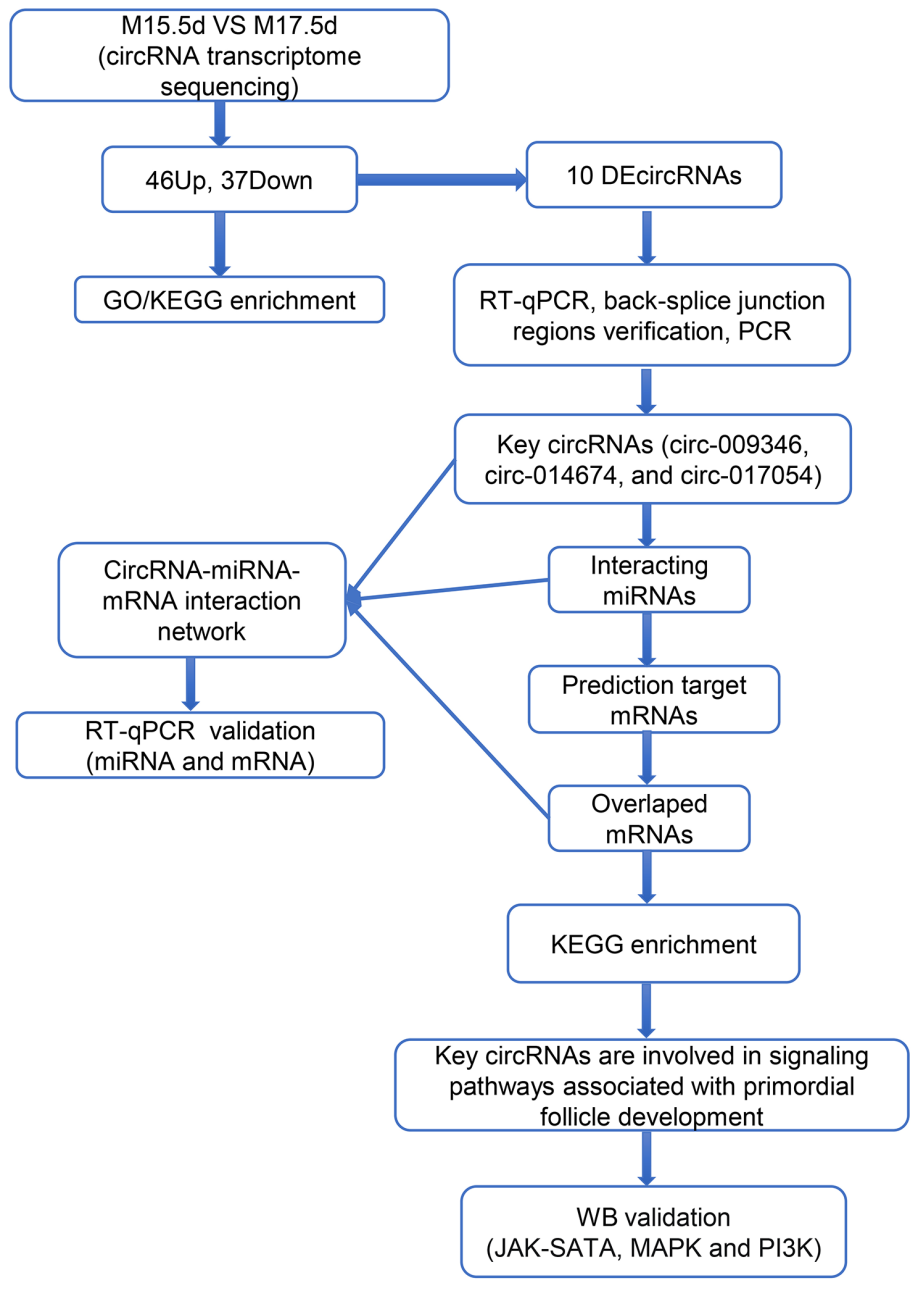
The fowchart of experimental procedure. M15.5d: 15.5 dpc mouse embryonic ovary; M17.5d: 17.5 dpc mouse embryonic ovary

**Fig. 2:**
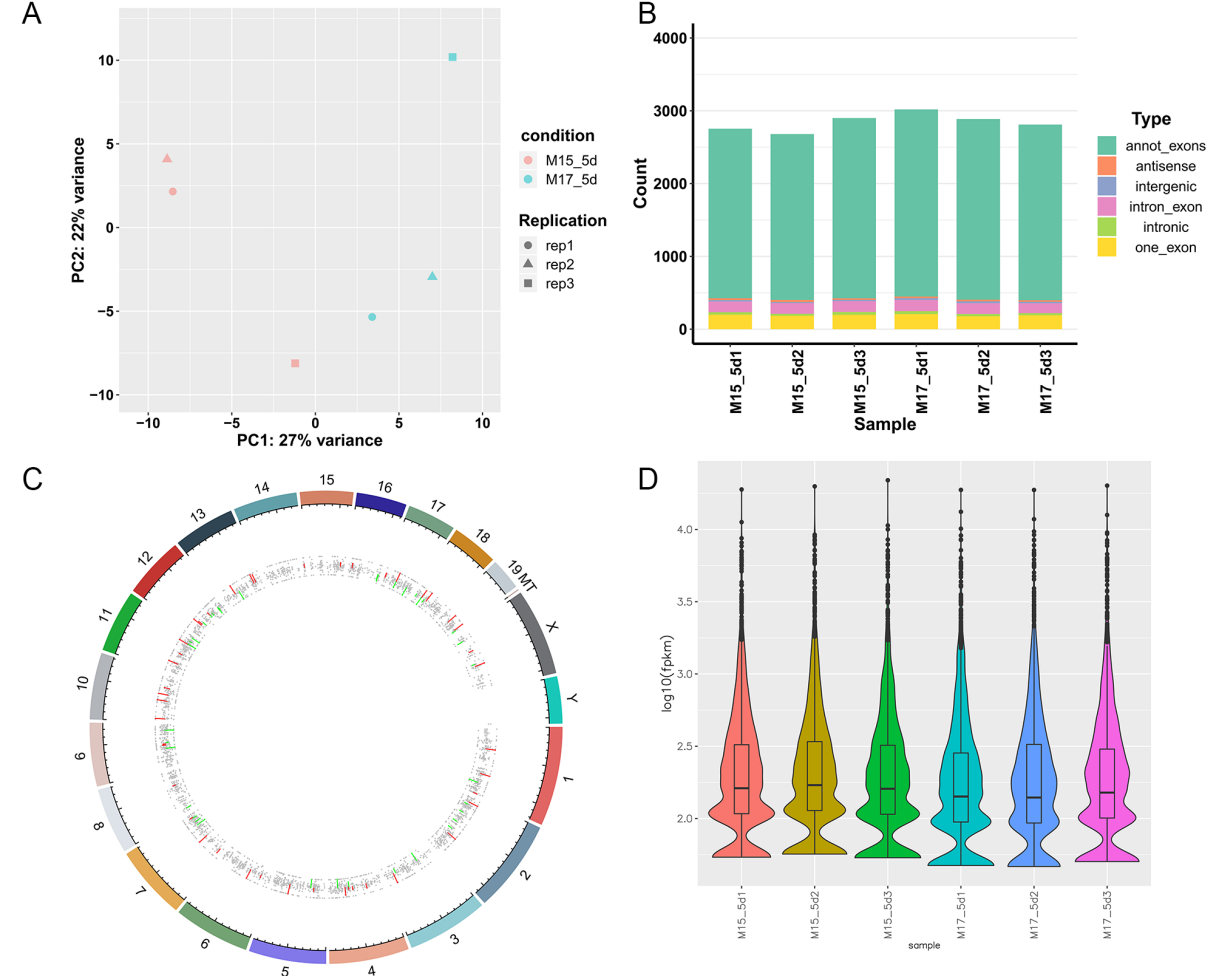
Global analysis of sequencing circRNAs. (**A**) Principal component analysis (PCA). (**B**) Distribution of circRNAs types in each sample. (**C**) Distribution of circRNAs expression in each chromosome. Red represents up-regulated genes and green represents down-regulated genes. (**D**) Statistical results of circRNA expression in each sample. The horizontal line in the middle of the box was the median, the upper and lower edges of the box were 75%, and the upper and lower limits were 90%. The external shape is a kernel density estimate

To determine the differential expression of circRNAs in the ovaries of 15.5 dpc and 17.5 dpc mice, the ovaries of mice in these two stages were collected for sequencing and analysis. The flowchart for this study is shown in Fig. 1. Six different libraries were pooled based on the amount of targeted data and sequenced on the Illumina HiSeq platform. After filtering the sequencing data, more than 5.9 million clean reads and 89 million clean reads (bp) were obtained from each library. The percentages of clean reads and clean data were both over 73%. The filtered reads were compared to the reference genome using HISAT2 software. The total mapped percentages were close to 90%, the multiple-mapped percentage was below 20% and the uniquely mapped percentage was above 80%, indicating that the reference genome was properly selected and that there was no pollution in the experiments (Table 1). To verify the reproducibility of the sequencing data, three sets of each sample were collected. High sample similarity was found by principal component analysis (PCA) (Fig. 2A), indicating that reliable sequencing data was collected.

After statistical analysis, we found that the distribution of circRNA types in 15.5 dpc and 17.5 dpc samples was similar, and these samples mainly included exon circRNAs, with only a small amount of intron and unannotated intergenic region circRNAs (Fig. 2B). The circRNA density statistics of each chromosome showed that circRNAs were distributed on chromosomes 1 to X, with the most circRNAs on chromosome 2 and the fewest circRNAs on chromosome 19. Furthermore, we did not observe distribution of circRNAs on the Y chromosome, indicating that the samples we collected were all from female mice (Fig. 2C, Supplemental Figure S1). The expression level of circRNA in each sample was relatively uniform, with the majority of circRNAs having medium expression and a small portion of circRNAs having either low expression or high expression (Fig. 2D).

**Table 1:** Summary of reads mapped to the mouse genome

Sample	Clean Reads (No)	Clean Data (bp)	Clean Reads (%)	Clean Data (%)	Total-Mapped (%)	Multiple-Mapped (%)	Uniquely-Mapped (%)
M15_5d1	64,344,420	9,651,663,000	73.53	75.53	56,443,342 (87.72%)	7,919,168 (14.03%)	48,524,174 (85.97%)
M15_5d2	63,913,596	9,587,039,400	73.88	73.88	57,014,569 (89.21%)	9,191,966 (16.12%)	47,822,603 (83.88%)
M15_5d3	59,640,132	8,946,019,800	73.2	73.2	52,744,730 (88.44%)	10,466,432 (19.84%)	42,278,298 (80.16%)
M17_5d1	63,110,866	9,466,629,900	74.07	74.07	56,501,249 (89.53%)	11,249,485 (19.91%)	45,251,764 (80.09%)
M17_5d2	60,128,982	9,019,347,300	75.92	75.92	53,828,630 (89.52%)	10,179,150 (18.91%)	43,649,480 (81.09%)
M17_5d3	62,144,486	9,321,672,900	73.83	73.83	55,175,488 (88.79%)	10,771,501 (19.52%)	44,403,897 (81.48%)

### Overall analysis of differentially expressed circRNAs

**Fig. 3:**
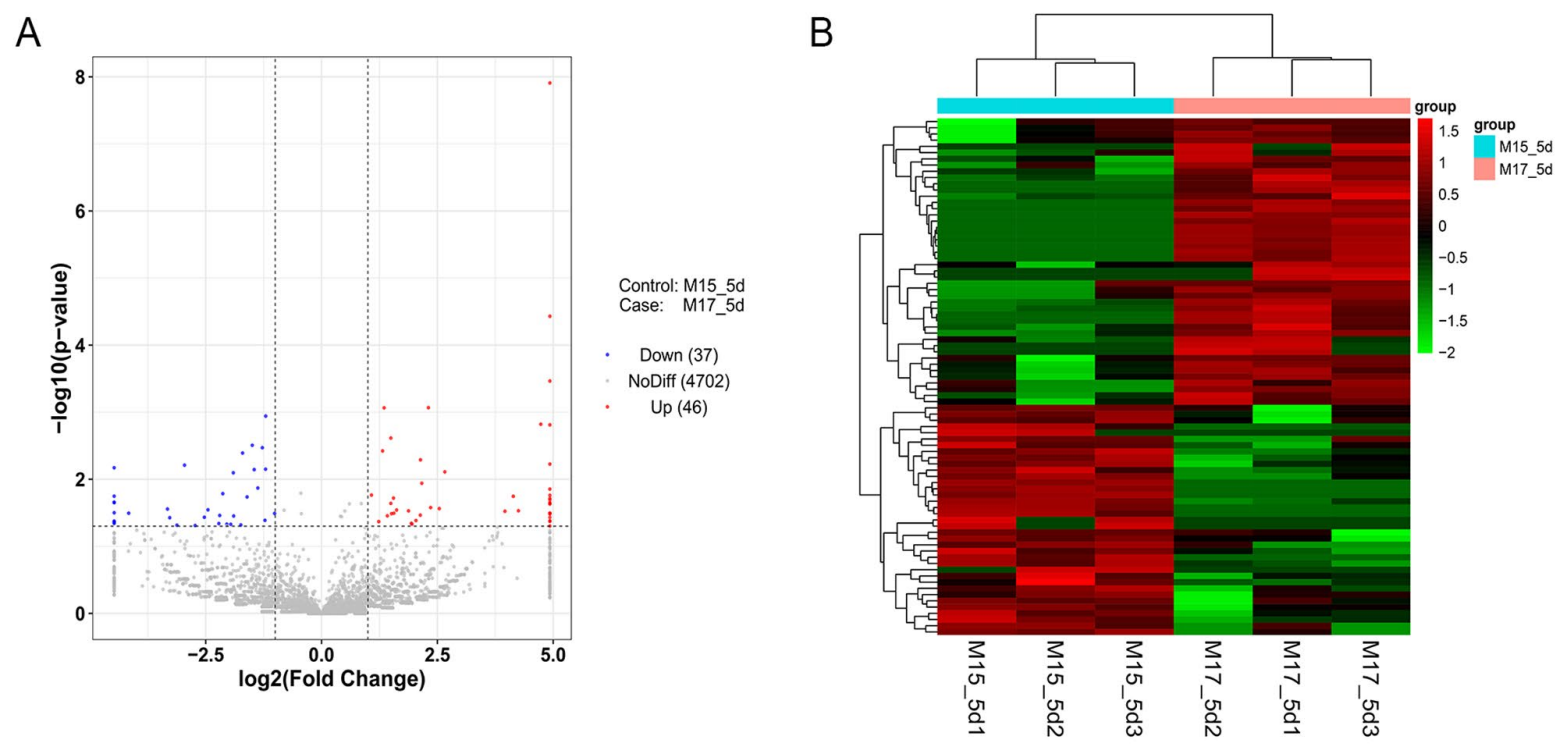
The total differentially expressed circRNAs. (**A**) The red dots indicated that circRNAs were up-regulated in this group, the blue dots indicated that circRNAs were down-regulated in this group, and the gray dots indicated that circRNAs were not differentially expressed. (**B**) Clustered heat map of differentially expressed circRNAs. The columns and rows in the heat maps represent samples and circRNAs, respectively. Sample names are displayed below the heat maps

A total of 4785 circRNAs in mouse embryonic ovaries were identified in this experiment (Supplemental Table S2). Of these circRNAs, 46 had upregulated expression and 37 had downregulated expression at 17.5 dpc compared with that at 15.5 dpc (Fig. 3A, Supplemental Table S3). Furthermore, we selected these differentially expressed circRNAs for hierarchical clustering analysis and found that the 15.5 dpc group was different from the 17.5 dpc group, indicating the consistency of the genetic backgrounds of the samples in each group (Fig. 3B). Ten circRNAs with significantly upregulated and downregulated expression were selected, as shown in Table 2.

**Table 2:** Biological information regarding the top 10 up-regulated and down-regulated circRNAs

Category	Gene ID	Location on Chromosome	FC	*P*-value	SourceGene
Up	circ-018161	6	Inf	1.29E-08	*Grid2*
	circ-001704	-	Inf	3.79E-05	*-*
	circ-019622	13	30.4465	3.53E-04	*Fars2*
	circ-020406	4	4.9429	8.79E-04	*Tmeff1*
	circ-021356	8	2.5489	8.81E-04	*Nfix*
	circ-014674	13	26.5265	1.56E-03	*Arhgef28*
	circ-013111	2	Inf	1.59E-03	*Fam227b*
	circ-014838	4	2.8201	2.50E-03	*Focad*
	circ-002297	6	2.4895	3.87E-03	*Ube2h; Zc3hc1*
	circ-011922	12	4.3924	5.20E-03	*Snx13*
Down	circ-001376	18	0.4336	0.0012	*Zeb1*
	circ-007367	12	0.3545	0.0032	*Rps6ka5*
	circ-017185	12	0.4124	0.0034	*Fut8*
	circ-022227	18	0.3068	0.0041	*Wdr33*
	circ-012633	7	0.1286	0.0062	*Smg1*
	circ-017054	18	0.0449	0.0069	*Asxl3*
	circ-022253	X	0.4329	0.0072	*Zfx*
	circ-008131	9	0.3650	0.0073	*Rfx7*
	circ-009944	19	0.2670	0.0081	*Cacul1*
	circ-015221	12	0.3853	0.0137	*Tc2n*

### Functional enrichment analysis of host genes of differentially expressed circRNAs

**Fig. 4:**
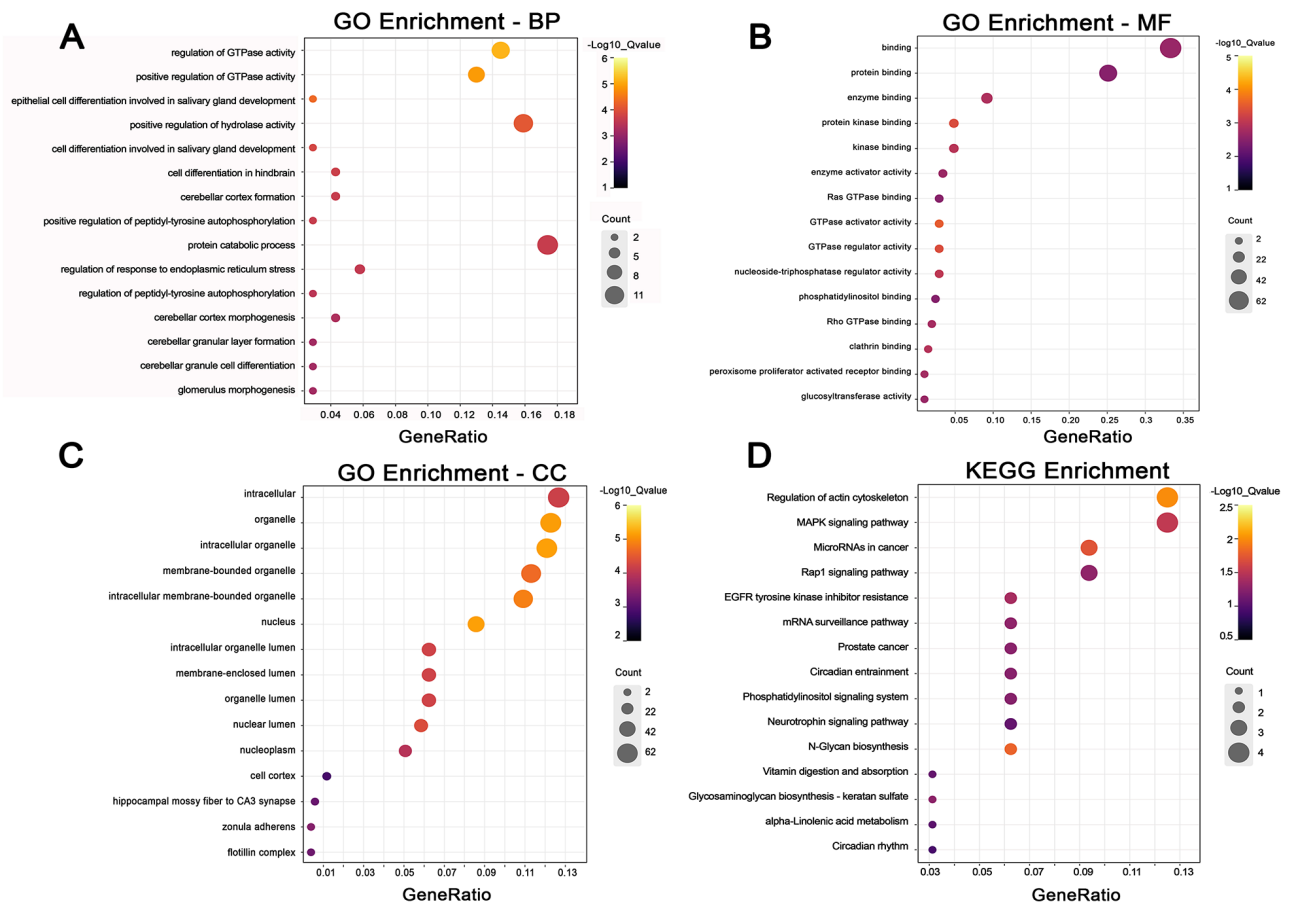
GO and KEGG pathway analysis for host genes of differentially expressed circRNAs. (**A**) Top 15 GO terms in biological processes. (**B**) Top 15 GO terms in molecular function. (**C**) Top 15 GO terms in cell component. (**D**) The top 15 pathways of KEGG enrichment

**Fig. 5:**
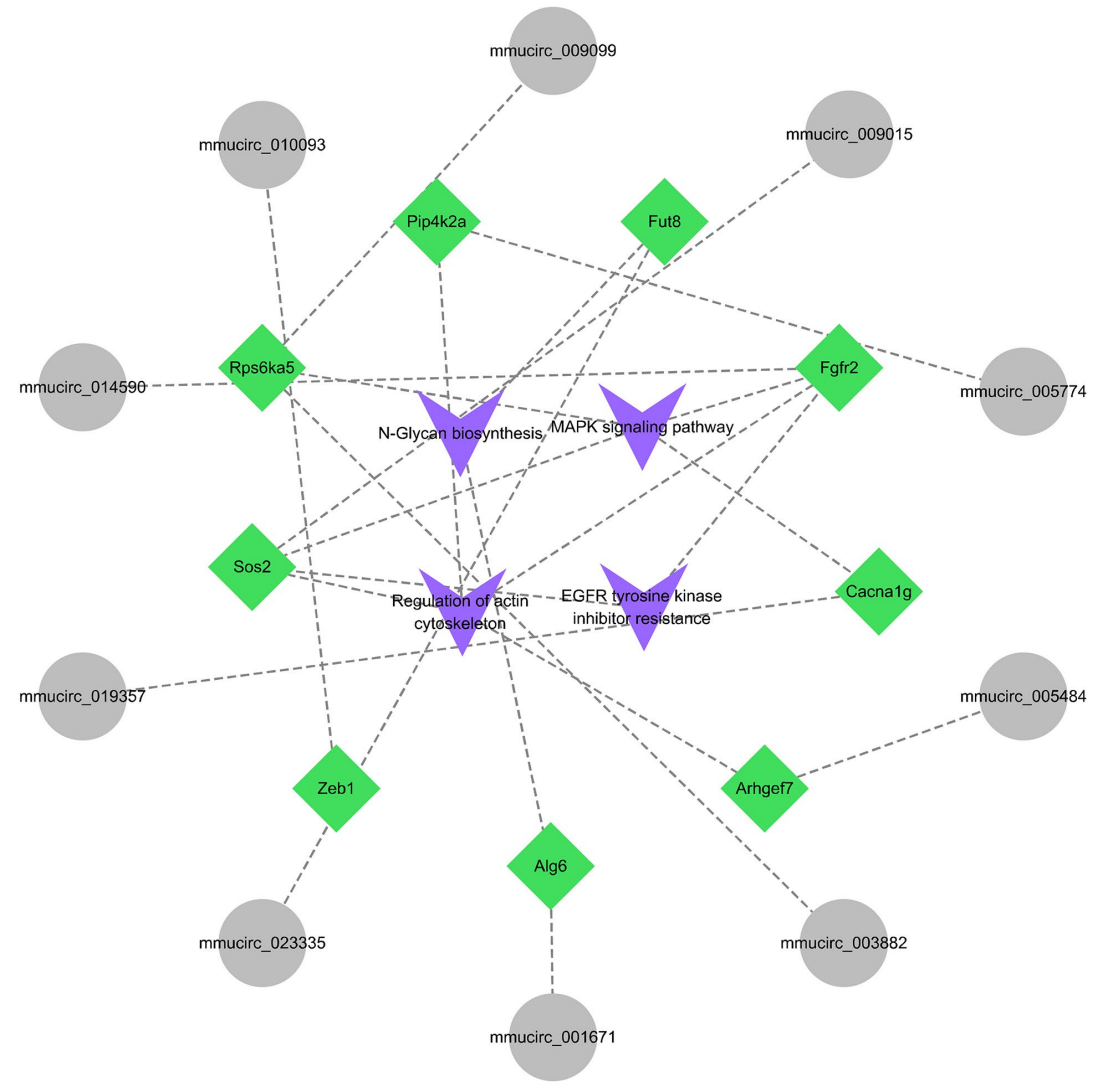
Significant enrichment pathway and enrichment gene visualization. Blue represents four significantly enriched pathways, green bands represent enriched genes, and gray represents circRNAs

GO and KEGG enrichment analyses were performed on the host genes of differentially expressed circRNAs. A total of 146 GO items were significantly enriched (*P* < 0.01) (Supplemental Table S4). The GO analysis results were classified according to molecular function (MF), biological process (BP) and cell component (CC). Thirty-eight significant enrichment items for MF, 78 significant enrichment items for BP, and 30 significant enrichment items for CC were obtained. In each GO classification, the top 15 GO terms were selected and displayed (Fig. 4A, B and C). The results showed enrichment in GO terms including organelles, nuclei and the regulation of enzyme activities. The top 15 pathways based on KEGG enrichment are shown in Fig. 4D. Interestingly, in these pathways, we found four important signalling pathways related to primordial follicle development, including the MAPK signalling pathway, actin cytoskeleton regulation, N-glycan biosynthesis and EGFR tyrosine kinase inhibitor resistance. Meanwhile, we selected the four significantly enriched pathways and their abundant genes for visualization using Cytoscape (Fig. 5). These findings suggest that the identified differentially expressed circRNAs may be involved in the regulation of primordial follicle development.

### Identification of key circRNAs

**Fig. 6:**
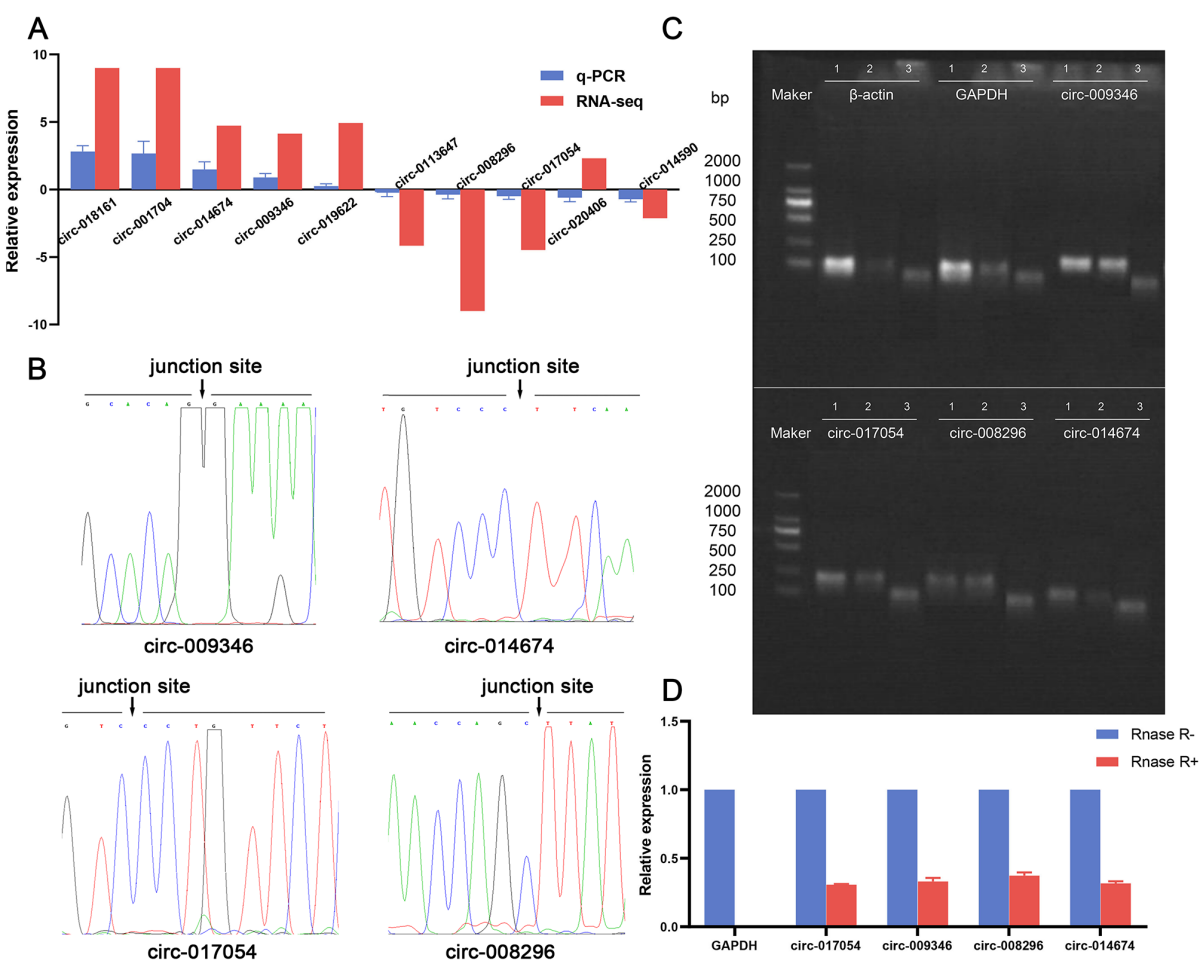
Identification of circRNAs associated with primordial follicle development. (**A**) The differentially expressed circRNAs were verified by qRT-PCR. (**B**) The back-splice junction regions verification of circRNAs. The red arrow represents the post-specification splice site. (**C**) The total RNA of ovary treated with or without RNase R was detected by nucleic acid electrophoresis after PCR. circ-009346, circ-014674, circ-017054 and circ-008296 were detected respectively. GAPDH was used as internal control, and β-actin was used as linear control. The first lane was treated without RNase R, the second lane was treated with RNase R, and the third lane was a negative control. (**D**) qRT-PCR was used to verify the enzyme digestion protection experiment. GAPDH was used as an internal reference, β-actin was used as a linear control. All experiments were repeated more than three times

First, we selected 10 differentially expressed circRNAs from the 83 differentially expressed circRNAs for qRT-PCR verification. The results showed that the expression of nine circRNAs (circ-019622, circ-018161, circ-009346, circ-008296, circ-013647, circ-014674, circ-017054, circ-014590 and circ-001704) was in agreement with the sequencing data, with the exception of circ-020406 (Fig. 6A). Next, we designed divergent primers that specifically amplified the back-splice junction regions of these nine circRNAs. Among the 9 differentially expressed circRNAs, we successfully amplified the back-splice junction regions of 4 circRNAs (circ-009346, circ-014674, circ-017054 and circ-008296) (Fig. 6B). The sequence information of these regions was consistent with the information obtained by sequencing, which indicated that these four circRNAs are indeed circular. Furthermore, the resistance of the four circRNAs to RNase R digestion was examined. These four circRNAs were resistant to RNase R, whereas the internal controls β-actin and GAPDH were sensitive to RNase R (Fig. 6C and D). This result further confirmed that these four circRNAs were resistant to enzymatic hydrolysis.

### Dynamic expression of key circRNAs

**Fig. 7:**
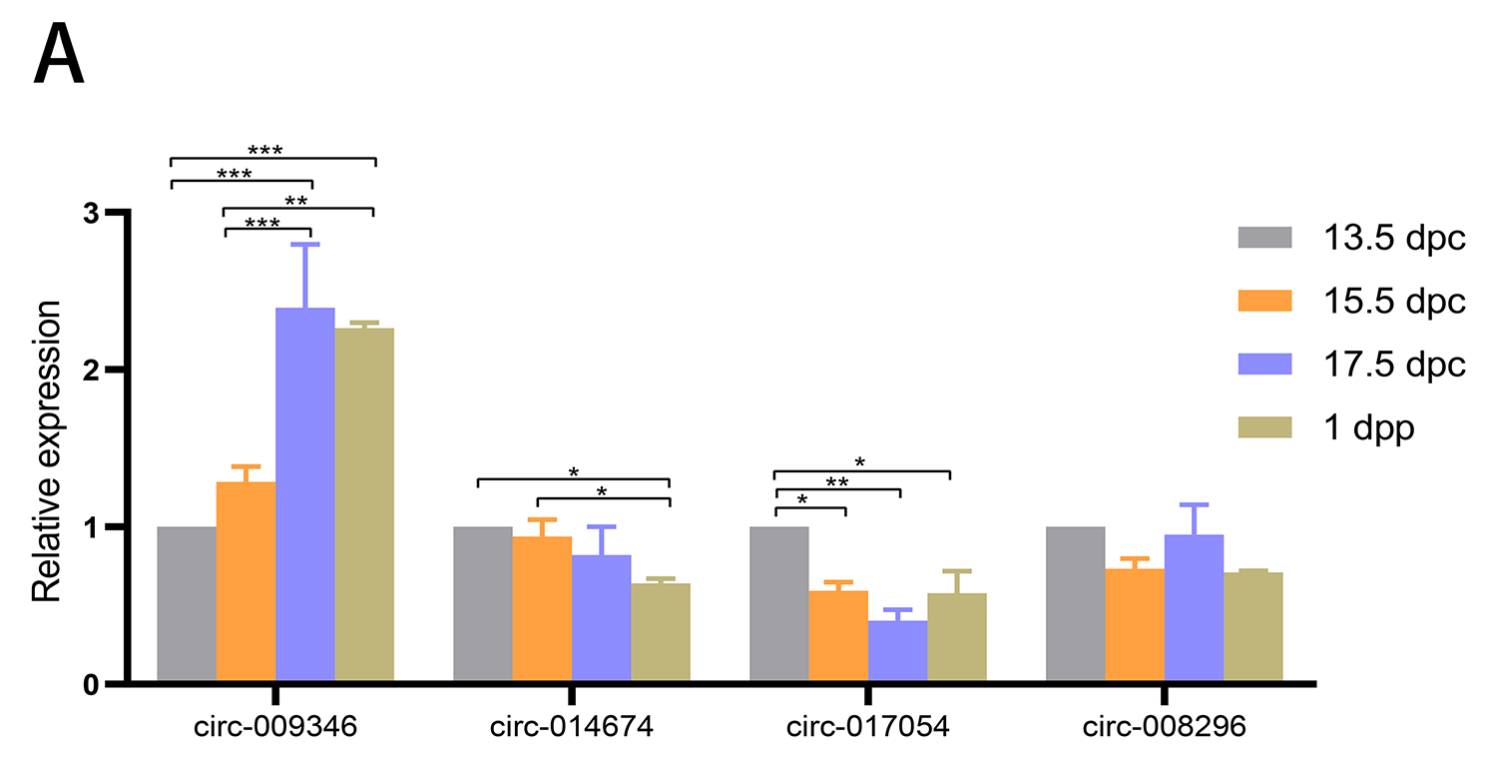
The dynamic expression of circRNAs in ovaries of mice at different developmental stages was detected by qRT-PCR (*P < 0.05, **P < 0.01, ***P < 0.001). All experiments were repeated more than three times

We provided information on four circRNAs with antienzyme properties in Table 3 and analysed the dynamic expression of these four key circRNAs in the ovaries of mice at different developmental stages (13.5 dpc, 15.5 dpc, and 17.5 dpc in the embryonic stage and 1 dpp in the neonatal stage) by qRT-PCR. The results showed that the expression levels of three circRNAs (circ-009346, circ-014674 and circ-017054) were significantly altered in the ovaries of mice at different stages of development (Fig. 7). These results suggested that circ-009346, circ-014674 and circ-017054 may be essential for primordial follicle development.

**Table 3:** The basic characteristics of key circRNAs

CircRNA	Chromosome	Length (nt)	Host gene	Description
circ-009346	Chr13	705	*Nln*	Neurolysin
circ-014674	Chr13	442	*Arhgef28*	Rho guanine nucleotide exchange factor
circ-017054	Chr18	422	*Asxl3*	Transcriptional regulator
circ-008296	Chr3	945	*Nbea*	Neurobeachin

### CircRNA-miRNA-mRNA network associated with primordial follicle development

**Fig. 8:**
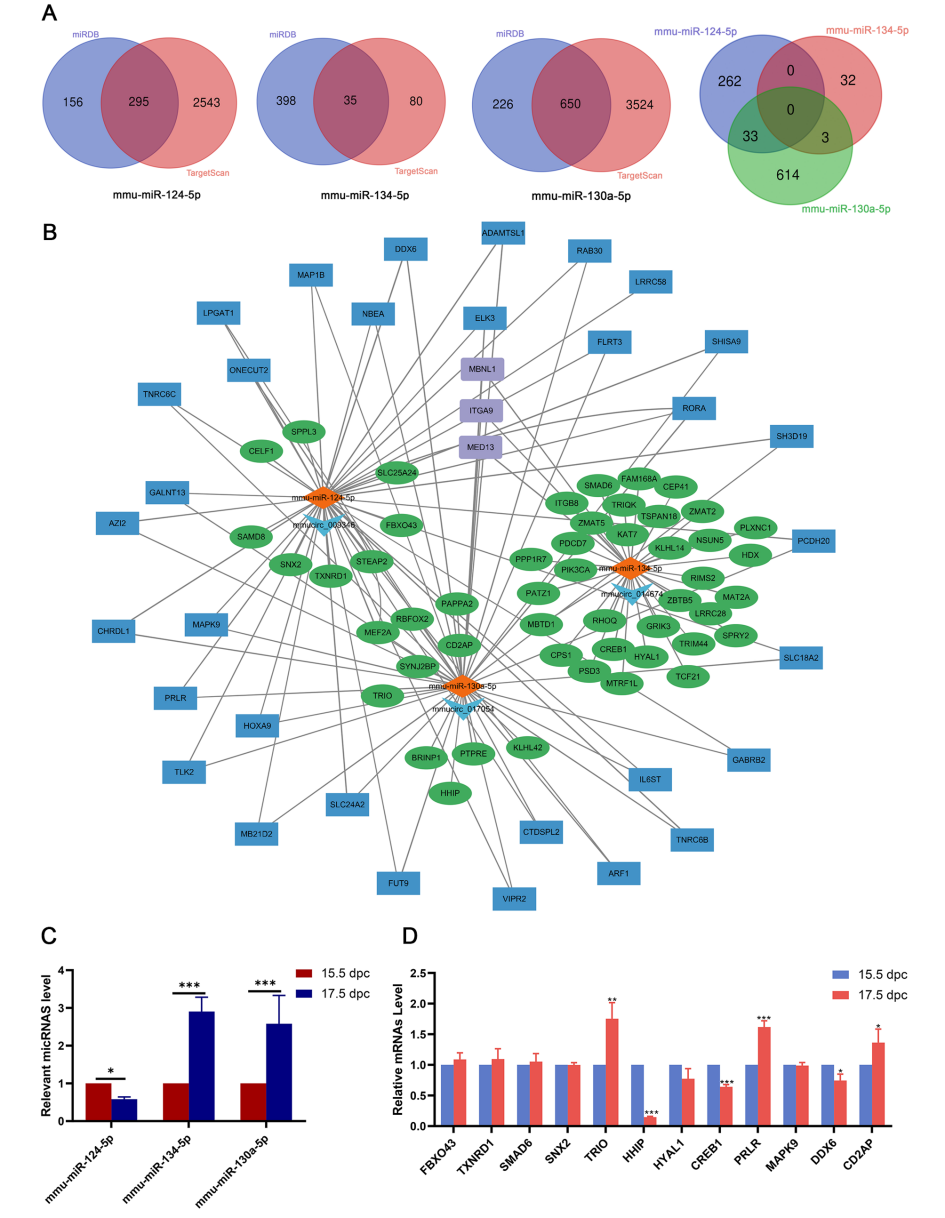
circRNA-miRNA-mRNA network associated with primordial follicle development. (**A**) The number of mRNA targeting miRNAs in the database and their overlap with each other. (**B**) The circRNA-miRNA-mRNA interaction network. The light blue V node represents circRNA, the orange diamond node represents the miRNA interacting with circRNA, the green node represents the mRNA obtained from the database, and the purple square node represents the mRNA overlaps between mmu-miR-134-5p and mmu-miR-130-5p. The blue square nodes represent mRNA overlapped by mmu-miR-124-5p and mmu-miR-130-5p. (**C**) Validation of micRNAs expression. (**D**) Validation of mRNAs expression. All experiments were repeated more than three times (*P < 0.05, **P < 0.01, ***P < 0.001)

Next, we used miRanda software to predict potential target genes of the miRNAs that interact with circ-009346, circ-014674 and circ-017054 (mmu-miR-124-5p, mmu-miR-134-5p, and mmu-miR-130a-5p). By comparing the predicted results of two online databases, miRDB and TargetScan, possible target mRNAs were identified, and some data were selected to generate a circRNA-miRNA-mRNA network map (Fig. 8A and B). The results showed that there were 295, 35 and 650 overlapping target genes predicted by the miRDB and TargetScan online databases for mu-mir-124-5p, mu-mir-134-5p and mu-mir-130a-5p, respectively. There were 33 overlapping target genes of mu-mir-124-5p and mu-mir-130a-5p and 3 overlapping target genes of mu-mir-134-5p and mu-mir-130a-5p. The qRT-PCR results demonstrated a significant decrease in the expression of mmu-miR-124-5p and a significant increase in the expression of mmu-miR-134-5p and mmu-miR-130a-5p in the embryonic ovary of 17.5 dpc samples compared to 15.5 dpc samples. Twelve important mRNAs were further selected from the circRNA-miRNA-mRNA network map for validation by qRT-PCR. TRIO, PRLR, and CD2AP showed significantly upregulated expression, HHIP, CREB1, and DDX6 showed significantly downregulated expression, and the other mRNAs (FBXO43, TXNRD1, SMAD6, SNX2, HYAL1, and MAPK9) did not show significant changes in expression. (Fig. 8C and D).

### Functional analysis of key circRNAs

**Fig. 9:**
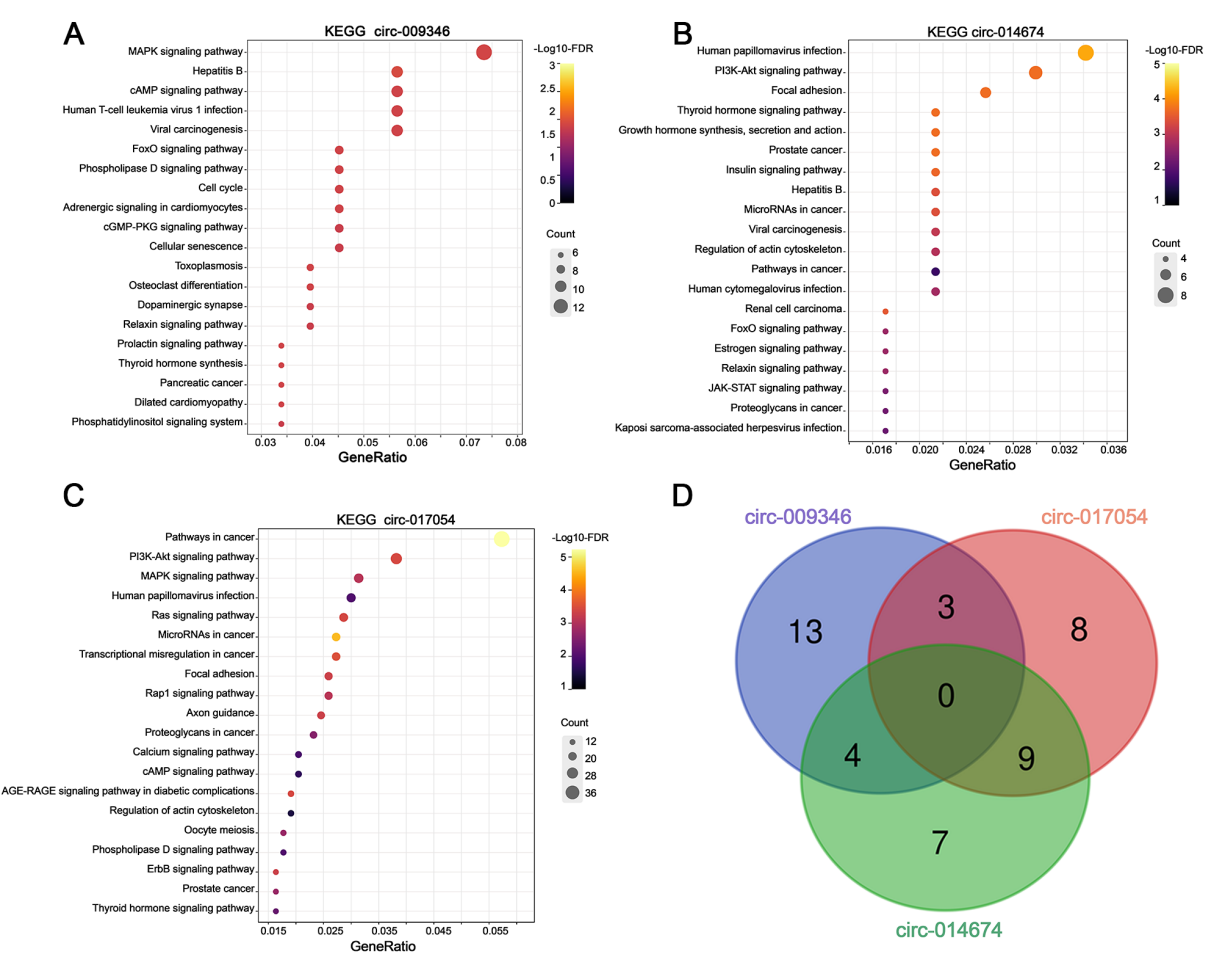
KEGG enrichment analysis of the interaction mRNAs for key circRNAs. (**A**) KEGG enrichment of the interaction mRNAs for circ-009346. (**B**) KEGG enrichment of the interaction mRNAs for circ-014674. (**C**) KEGG enrichment of the interaction mRNAs for circ-017054. (**D**) The Venn diagram showed that the overlapped KEGG enrichment pathways of the interaction mRNAs for circ-009346, circ-014674 and circ-017054

To further explore the effect of key circRNAs on primordial follicle development, we performed KEGG analysis of the mRNAs of the circRNA-miRNA-mRNA interaction network and selected the top 20 pathways to display (Fig. 9A, B and C). The Venn diagram showed the overlapping KEGG enrichment pathways of the interacting mRNAs for circ-009346, circ-014674 and circ-017054 (Fig. 9D). The results showed that the interacting mRNAs of these three circRNAs were significantly enriched in pathways related to primordial follicle activation, such as the MAPK signalling pathway, the cAMP signalling pathway, and the PI3K signalling pathway. In addition, the interacting mRNAs of circ-014674 and circ-017054 were involved in growth hormone synthesis, the oestrogen signalling pathway, the calcium signalling pathway and oocyte meiosis.

### The potential mechanism of key circRNAs in primordial follicle development

**Fig. 10:**
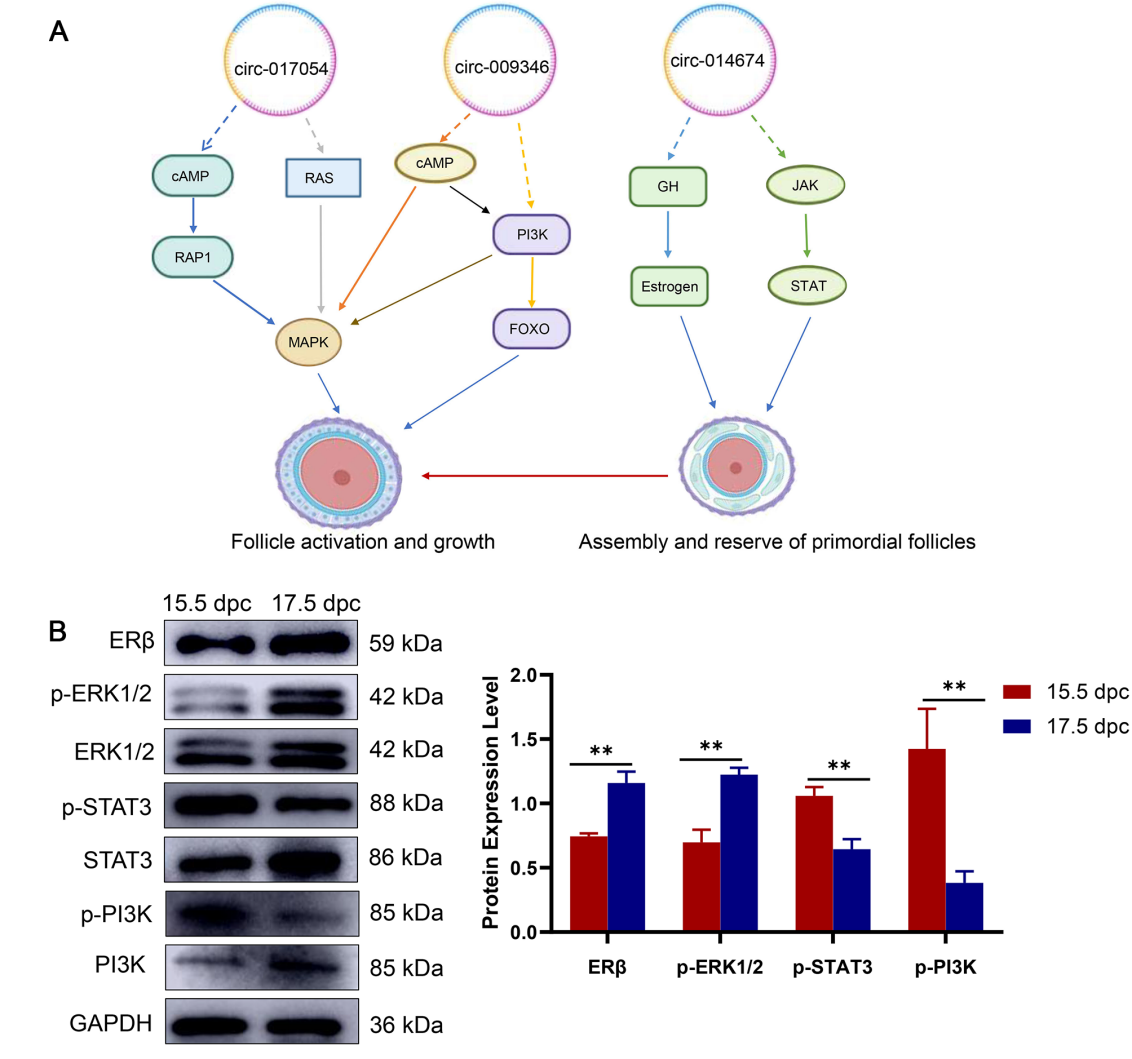
The potential mechanism of key circRNAs on primordial follicle development. (**A**) The schematic diagram of potential mechanism of key circRNAs in primordial follicle development. Dotted lines represent speculations, solid lines represent proven results. (**B**) MAPK, PI3K, oestrogen and JAK-STAT signaling pathways are involved in primordial follicle formation. All experiments were repeated more than three times (*P < 0.05, **P < 0.01, ***P < 0.001)

To understand the signalling pathways by which circRNAs impact primordial follicle development, we summarized the signalling pathways of interacting mRNAs of key circRNAs (circ-009346, circ-014674 and circ-017054) based on our studies and previous reports (Fig. 10A). We found that the key circRNAs may act through the MAPK, PI3K, oestrogen and JAK-STAT signalling pathways. Therefore, we investigated the expression of key proteins in the MAPK, PI3K, oestrogen, and JAK-STAT signalling pathways in 15.5 dpc and 17.5 dpc embryonic mouse ovaries using western blotting analysis. The results showed that the expression of oestrogen receptor and p-ERK1/2, key proteins in the oestrogen and MAPK signalling pathways, was significantly upregulated in the ovaries of 17.5 dpc embryos compared to that in the ovaries of 15.5 dpc embryos. The expression of the key proteins of the PI3K and JAK-STAT signalling pathways, p-STAT3 and p-PI3K, was significantly downregulated (Fig. 10B). This suggests that these signalling pathways may be involved in primordial follicle formation and activation. Based on these results, we speculated that circ-017054 was mainly involved in the activation and growth of primordial follicles through the MAPK signalling pathway. Meanwhile, circ-009346 was also involved in the activation and growth of primordial follicles. Circ-009346 was mainly involved in the activation and growth of primordial follicles through the MAPK and PI3K signalling pathways, and circ-014674 was involved in primordial follicle assembly and reserve mainly via the growth hormone (GH)-oestrogen and JAK-SATA signalling pathways.

## Discussion

The follicle is the basic unit of reproduction in female mammals. Follicles not only provide a stable microenvironment for oocyte development but also secrete hormones to regulate physiological activities related to reproduction [23, 24]. In most mammals, the primordial follicle pool is established before and after birth and cannot be renewed. The number of primordial follicles determines the reproductive lifespan of female mammals [25]. In mouse embryos at 15.5 dpc, a large number of syncytia are formed, and most oocytes start meiosis [6]. In mice, the syncytium ruptures at 17.5 dpc, initiating the formation of primordial follicles and activating the first batch of primordial follicles [7]. Therefore, 15.5 dpc and 17.5 dpc in the mouse embryonic stage are key stages of primordial follicle development [26, 27]. In this study, mouse ovaries at 15.5 dpc and 17.5 dpc were selected for circRNA sequencing, and a total of 4785 circRNAs were obtained. Among these 4785 circRNAs, 83 differentially expressed circRNAs were found. The expression of 46 circRNAs was upregulated, and that of 37 circRNAs was downregulated. Since the periods of 15.5 dpc and 17.5 dpc are important for the development of primordial follicles in mice, we hypothesized that these 83 differentially expressed circRNAs might be closely related to the development of primordial follicles.

GO and KEGG enrichment analyses were performed using the host genes of the differentially expressed circRNAs. GO analysis showed that the host genes of the differentially expressed circRNAs were mainly involved in the regulation of enzyme activity and cell differentiation in cells. KEGG enrichment analysis [28, 29] revealed that the MAPK signalling pathway, regulation of the actin cytoskeleton, N-glycan biosynthesis and EGFR tyrosine kinase inhibitor resistance were significantly enriched. Furthermore, appropriate activation of the MAPK signalling pathway can facilitate the activation and development of ovarian follicles in vitro; thus, this pathway plays a regulatory role in the development of primordial follicles [30]. The TGF-β signalling pathway can affect the development of primordial follicles [31], and the actin cytoskeleton regulatory pathway can regulate the TGF-β signalling pathway [32]; thus, the actin cytoskeleton regulatory pathway also plays a regulatory role in the development of primordial follicles. In addition, N-glycans play a regulatory role in follicles at different stages of development [33]. Taken together, the results of our analysis indicated that these differentially expressed circRNAs are indeed closely related to the development of primordial follicles.

Next, ten circRNAs were selected to verify the accuracy of sequencing, and four circRNAs (circ-009346, circ-014674, circ-017054 and circ-008286) were identified. We found that circ-009346, circ-014674 and circ-017054 had significant changes in expression during the development of ovaries. Because circ-009346, circ-014674 and circ-017054 are likely to be the three key circRNAs involved in primordial follicle development in mice, we used miRanda software to predict miRNAs corresponding to these circRNAs and predicted mRNAs corresponding to the miRNAs using the miRDB and TargetScan online databases. The circRNA-miRNA-mRNA interaction network was constructed using Cytoscape software based on a portion of mRNA data that was in agreement based on both databases. From the interaction network diagram, we found that mmu-miR-124-5p and mmu-miR-134-5p, corresponding to circ-009346 and circ-014674, overlap with mmu-miR-130a-5p, corresponding to circ-017054, to varying degrees. This suggests that these circRNAs may have some of the same functions in primordial follicle development. We validated the target miRNAs and mRNAs by qRT-PCR and found that mmu-miR-124-5p, HHIP, CREB1, and DDX6 showed significantly decreased expression at 17.5 dpc, while mmu-miR-134-5p, mmu-miR-130a-5p, TRIO, PRLR, and CD2AP showed significantly upregulated expression. We hypothesized that mmu-miR-124-5p, mmu-miR-134-5p, mmu-miR-130a-5p, TRIO, PRLR, CD2AP, HHIP, CREB1 and DDX6 are involved in primordial follicle formation and activation. Next, we performed KEGG enrichment analysis of these interacting mRNAs based on the circRNA-miRNA-mRNA interaction network and summarized the pathways that circRNAs might impact in relation to the primordial follicle. We found that circ-017054 was significantly enriched in the cAMP signalling pathway, the RAP1 signalling pathway, the RAS signalling pathway and the MAPK signalling pathway. It is well known that follicles are mainly composed of granulosa cells and oocytes, and the development of granulosa cells and oocytes both affect the development of follicles [34]. MAPK signalling pathways include the ERK, JNK/SAPK and P38 MAPK signalling pathways [35]. Studies have shown that the cAMP-RAP1-P38 MAPK signalling pathway is activated in immature granulosa cells [36], and the RAS-MAPK signalling pathway is involved in follicle growth [37]. Therefore, we speculate that circ-017054 may impact the growth of primordial follicles through the cAMP-RAP1-MAPK and RAS-MAPK signalling pathways. In addition, circ-009346 was significantly enriched in the cAMP signalling pathway, the PI3K signalling pathway, the FOXO signalling pathway and the MAPK signalling pathway. Some studies have found that cAMP can activate MAPK in follicles at all levels and promote follicle growth [38]. The PI3K-PKC-MAPK and cAMP-PKC-PI3K signalling pathways are involved in the activation and growth of primordial follicles [39, 40]. The PI3K pathway consists of AKT and FOXO, and mouse experiments have demonstrated that PI3K controls the activation of primordial follicles [41]. Our western blotting experiments confirmed the participation of the MAPK, PI3K, oestrogen, and JAK-STAT signalling pathways in the development of primordial follicles. Therefore, we speculate that circ-009346 may impact the activation and growth of primordial follicles through the cAMP-MAPK, PI3K-PKC-MAPK, cAMP-PKC-PI3K and PI3K-FOXO signalling pathways. Circ-014674 was significantly enriched in the growth hormone (GH), oestrogen and JAK-SATA signalling pathways. Experiments have shown that growth hormone (GH) stimulates the production of oestrogen, which plays an important role in primordial follicle assembly [42, 43]. The JAK-SATA signalling pathway can help maintain primordial follicle reserves [44]. Similarly, we speculate that circ-014674 may be involved in the assembly and reserve of primordial follicles through the oestrogen and JAK-SATA signalling pathway.

However, our experiment does have some limitations. Although our study involved pathway prediction, we did not conduct in-depth studies on the predicted miRNAs, mRNAs and signalling pathways. In recent years, an increasing number of experiments have proven that circRNAs can be translated into peptides or proteins through a certain translation mechanism, and the construction of a circRNA-protein network during primordial follicle development would play a crucial role in revealing the mechanisms underlying primordial follicle development. However, due to the limitations of our study, we did not address how circRNAs affect these signalling pathways during primordial follicle development and thus affect primordial follicle development. In addition, we can use excellent computational models to predict the progress of subsequent circRNA research and its application in treatment of diseases associated with primordial follicle development [45–47]. For example, the mechanism of NLRP1b inflammatory vesicle signalling is revealed at a new level by new models [48], and the correlation between metabolites and disease is inferred by new algorithms [45]. New physical models have been proposed to describe high-valence mRNA-protein interactions, and this finding also provides a certain basis for future disease treatment and mechanism unravelling [49]. We can more deeply reveal the key role of circRNAs in primorval follicles by combining new models and computational methods in subsequent studies.

## Conclusions

In this study, 83 differentially expressed circRNAs were identified for the first time by RNA sequencing at 15.5 dpc and 17.5 dpc, which are critical periods of primordial follicle development in mouse embryonic ovaries. Of these, 46 circRNAs had upregulated expression and 37 circRNAs had downregulated expression in 17.5 dpc samples compared with 15.5 dpc samples. Our analysis indicated that these differentially expressed circRNAs are closely related to the development of primordial follicles. Furthermore, we identified three key circRNAs (circ-009346, circ-014674, and circ-017054) and mapped the circRNA-miRNA-mRNA network. Finally, we found that circ-014674 may participate in the assembly and reserve of primordial follicles through the oestrogen and JAK-SATA signalling pathways. Circ-009346 and circ-017054 may have similar functions and are involved in the activation and growth of primordial follicles through the MAPK and PI3K signalling pathways. These findings provide important references and targets for the development of primordial follicles. In the future, we will focus on the function and mechanism of these key circRNAs in primordial follicle development.

## Materials and methods

### Animal and ovary collection

In this experiment, 8 to 10-week-old ICR mice (purchased from Beijing Vital River Laboratory Animal Technology Co., Ltd.) were selected. The room temperature was maintained at 24–26 °C. The diet was free and the mice were housed under 12 h/12 h alternating light and dark. All mouse-related operations in this experiment were approved by the Animal Ethics and Welfare Committee of Ningxia University. Adult female and male mice were mated at a 1:1 ratio in the evening to induce pregnancy, followed by vaginal plug detection the next morning. Mice with vaginal plug detection were defined as 0.5 day post coitus (dpc) and considered 1 day postpartum (dpp) (Teng, et al. 2016) the second day after delivery.

All female mice were randomly divided into the following four groups: the 13.5 dpc embryonic ovary group, the 15.5 dpc embryonic ovary group, the 17.5 dpc embryonic ovary group, and the 1 dpp neonatal ovary group. All mice were sacrificed by cervical dislocation. After the mice in the embryonic ovary group were sacrificed, the abdomen was dissected to obtain the foetus in utero and sacrificed immediately, and the embryonic ovaries were collected. After neonatal ovary group mice were sacrificed, the ovaries of neonatal mice were collected. All collected ovaries were separated under a stereomicroscope to ensure ovarian integrity.

### RNA extraction

An appropriate amount of tissue was transferred to a 1.5 mL enzyme-free EP tube with 1.0 mL TRIzol (Hefei Bomei Biotechnology Co., LTD, BM1144); the samples were then pulverized with a high-speed low-temperature tissue lapping machine. The homogenate sample was placed at room temperature (15–30 °C) for 5–15 min to allow complete separation of the nucleic acid protein complex. For every 1.0 mL of TRIzol, 0.2 mL of chloroform was added, and the mixture was shaken vigorously and left at room temperature. Then, samples were centrifuged for 15 min at 12,000 r (2–8 °C). The above aqueous phase containing RNA was transferred to a new enzyme-free EP tube, and the same amount of isopropanol (Sangon Biotech, B422BA0020) was added and the samples were mixed upside down. The supernatant was discarded after centrifugation at 12,000 r for 15 min at 2–8 °C. The RNA precipitate was washed with 75% ethanol. Then, the samples were placed at room temperature, centrifuged at 2–8 °C at 5000 r/min for separation for 3 min, and the supernatant was discarded. Then, 10 µL DEPC (Sigma, D575810132) water was added and the samples were stored at 4 °C for later use.

### RNA sequencing

After RNA extraction, purification and library construction, the libraries were sequenced by next-generation sequencing (NGS) on the Illumina HiSeq sequencing platform. The raw data were filtered, and the clean data were aligned to the reference genome of the respective species. For unaligned sequences, anchor reads were obtained with 20 bp anchors. These anchor reads were realigned with the genome, and according to the alignment results, circRNAs were identified using find_circ. Finally, basic statistical analysis and quantitative and differential expression analyses were performed for circRNAs. CircRNAs with an adjusted [log2 (fold change)] > 1 and *P* < 0.05 or [log2 (fold change)] <-1 and *P* < 0.05 were identified as differentially expressed circRNAs.

### qRT-PCR analysis

Total RNA was reverse transcribed into cDNA, and qRT-PCR was performed using the qRT-PCR Starter Kit (RiboBio Co., Ltd, C11030-1) in a quantitative real-time PCR (qRT-PCR) instrument (QuantStudio TM3). The amplification reaction parameters were as follows: denaturation at 95 °C for 10 min, followed by annealing extension at 95 °C for 5 s, 60 °C for 30 s and 72 °C for 30 s for a total of 45 cycles. Fold changes in expression were calculated using the comparative delta-delta CT method (2^−ΔΔCt^) method with GAPDH as the internal control. All experiments were repeated three times, and the primers used in this experiment are listed in Supplemental Table S1.

### qRT-PCR analysis of miRNAs

Total RNA was synthesized into cDNA using a miRNA first strand cDNA synthesis kit (Sangon Biotech, #B532451), and qRT-PCR was performed on a real-time fluorescence quantitative PCR instrument using a miRNA fluorescence quantitative PCR kit (Sangon Biotech, #B532461). The amplification reaction parameters were as follows: predenaturation at 95 °C for 30 s, denaturation at 95 °C for 5 s, and annealing at 60 °C for 30 s for a total of 40 cycles. All experiments were repeated three times, and the primers used in this experiment are listed in Supplemental Table S1.

### Back-splice sequence detection of circRNAs

The circRNA back-splice sequences were amplified by RT-PCR. Furthermore, Sanger sequencing was performed to directly examine the RT-PCR product. If the sequence of the RT-PCR product was consistent with that obtained by previous RNA sequencing, it indicates that the circRNA was circular. The RT-PCR procedure was consistent with the qRT-PCR procedure for circRNA.

### RNase R digestion experiment for circRNAs

Total RNA samples (5 µg) of embryonic ovaries were divided into two sterilized PCR tubes, RNase R was added to one tube at 3 U/µg, and the other tube was used as a control. Both tubes were placed in a PCR instrument and heated at 37 °C for 30 min. PCR and qRT-PCR were performed to confirm the resistance of circRNAs to RNase R.

### Immunoblotting

The ovaries of mice at 15.5 dpc and 17.5 dpc were collected, and the total ovarian protein was extracted using the total protein extraction kit. The protein was separated by SDS-PAGE and transferred to a PVDF membrane. The membrane was blocked with 5% skim milk for 2 h and then incubated with antibodies overnight at 4 °C. The following primary antibodies were used: oestrogen receptor beta (ERβ) (1:1000; ABclonal; A2546); ERK1/2 (1:500; ABclonal; A4782); p-ERK1/2 (1:500; ABclonal; AP0485); STAT3 (1:500; ABclonal; A19566); p-STAT3 (1:500; ABclonal; AP0715); PI3K (1:1000; Proteintech; 60225-1-Ig); p-PI3K (1:1000; Cell Signaling Technology; 4228 S); and GAPDH (1:5000; Proteintech; 10494-1-AP). After staining with HRP-conjugated secondary antibody for 2 h, the proteins on the membrane were detected by Bio-Rad ChemiDoc MP. The blots were trimmed to enhance the clarity and succinctness of the images.

### Bioinformatics analysis

CircRNAs are produced from their host genes. Thus, analysis of host genes can provide valuable insights into the function of circRNAs. Gene Ontology (GO (http://geneontology.org/) was used to annotate and classify the host genes of differentially expressed circRNAs based on GO terms for biological process (BP), cellular component (CC), and molecular function (MF). Kyoto Encyclopedia of Genes and Genomes (KEGG; http://www.genome.jp/kegg/) pathway enrichment analysis was performed to identify significantly enriched metabolic pathways or signal transduction pathways in host genes compared with the whole genome background. *P* < 0.05 indicated that GO items or KEGG pathways of the host genes of differentially expressed circRNAs were significantly enriched. TargetScan **(**http://www.targetscan.org/mamm_31/) and miRDB (http://www.mirdb.org/) were used to predict the miRNA target genes. Functional analysis of the interactions of genes related to key circRNAs were obtained from STRING online software (https://string-db.org).

### Statistical analysis

All data were obtained from at least three independent experiments. GraphPad Prism 8 was used to analyse the data, which were expressed as the mean ± standard deviation (SD). One-way analysis of variance (ANOVA) was used to analyse the experimental data. A t test was used to analyse the difference between the two groups. *p* < 0.05 indicates a significant difference. The circRNA-miRNA-mRNA interaction network was visualized using Cytoscape software. Visualization of bubble plots was performed using the online software chiplot (https://www.chiplot.online).

### Electronic supplementary material

Below is the link to the electronic supplementary material.

Supplementary Material 1

Supplementary Material 2

Supplementary Material 3

Supplementary Material 4

Supplementary Material 5

Supplementary Material 6

Supplementary Material 7

Supplementary Material 8

Supplementary Material 9

Supplementary Material 10

Supplementary Material 11

Supplementary Material 12

Supplementary Material 13

Supplementary Material 14

Supplementary Material 15

## Data Availability

All data relevant to the study has been submitted to the SRA database, study accession number: PRJNA934979. The data is accessible at the following link: https://www.ncbi.nlm.nih.gov/sra/PRJNA934979.

## Abbreviations

PGCs: Primordial germ cells
CircRNA: Circular RNA
DPC: Day post coitus
DPP: Day postpartum
GO: Gene Ontology
BP: Biological process
CC: Cell component
MF: Molecular function
KEGG: Kyoto Encyclopedia of Genes and Genomes
TPM: Transcripts Per Million
cDNA: Complementary DNA
qRT-PCR: Quantitative Real-Time PCR
