# Supplementary figures and images for: Identification and analysis of key circRNAs in the mouse embryonic ovary provides insight into primordial follicle development
Source: BMC Genomics. 2024 Feb 3;25:139. doi: 10.1186/s12864-024-10058-y (PMC10837906; doi:10.1186/s12864-024-10058-y)

Supplementary Information

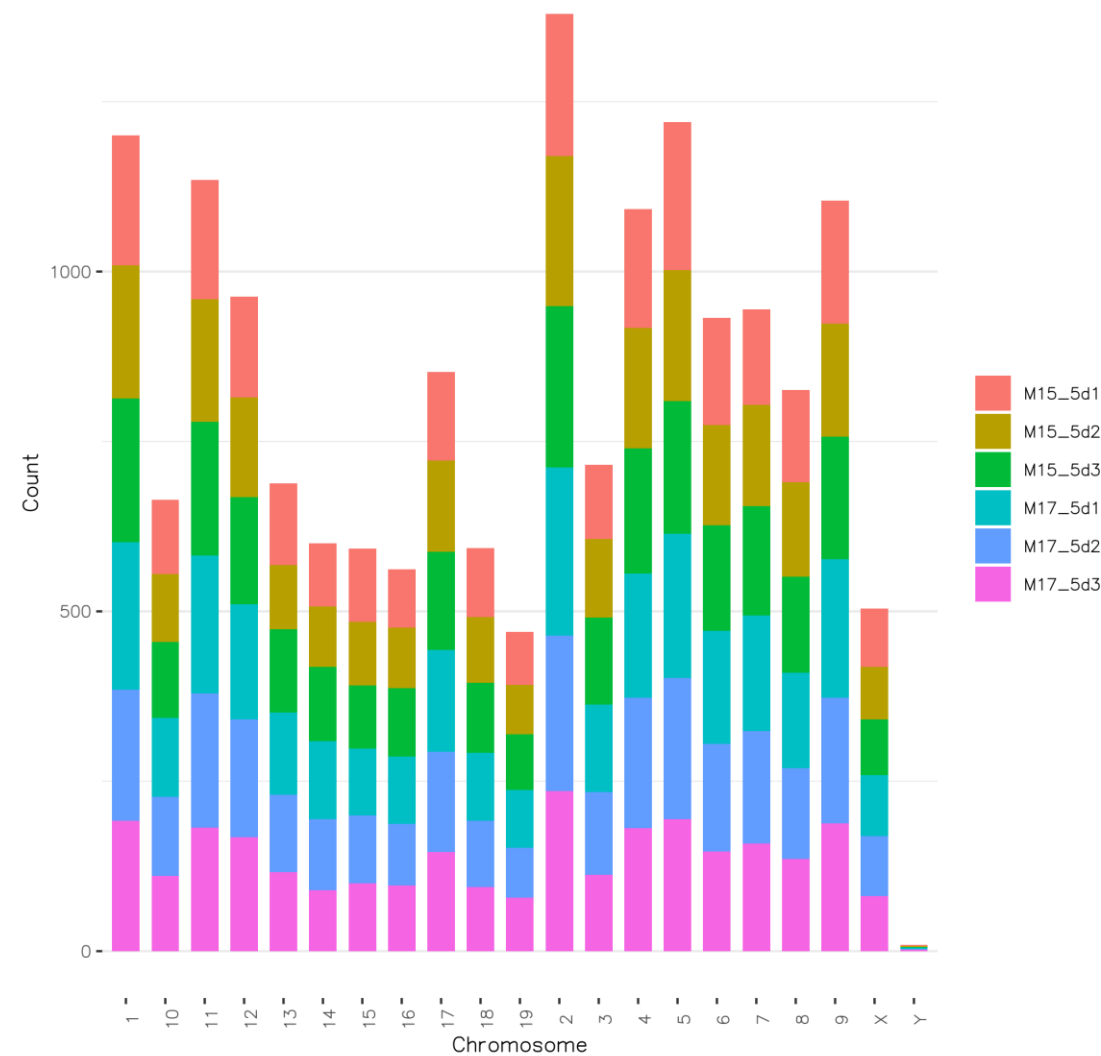

Additional file 1. Figure S1. Distribution of circRNA on chromosomes.

Supplement: Supplementary file 1 — Supplementary Material 1 [file 12864_2024_10058_MOESM1_ESM.pdf]
