# Supplementary material for: Identification and analysis of key circRNAs in the mouse embryonic ovary provides insight into primordial follicle development
Source: BMC Genomics. 2024 Feb 3;25:139. doi: 10.1186/s12864-024-10058-y (PMC10837906; doi:10.1186/s12864-024-10058-y)
Supplement: Supplementary file 14 — Supplementary Material 14 [file 12864_2024_10058_MOESM14_ESM.pdf]

# Original images 2: western blot

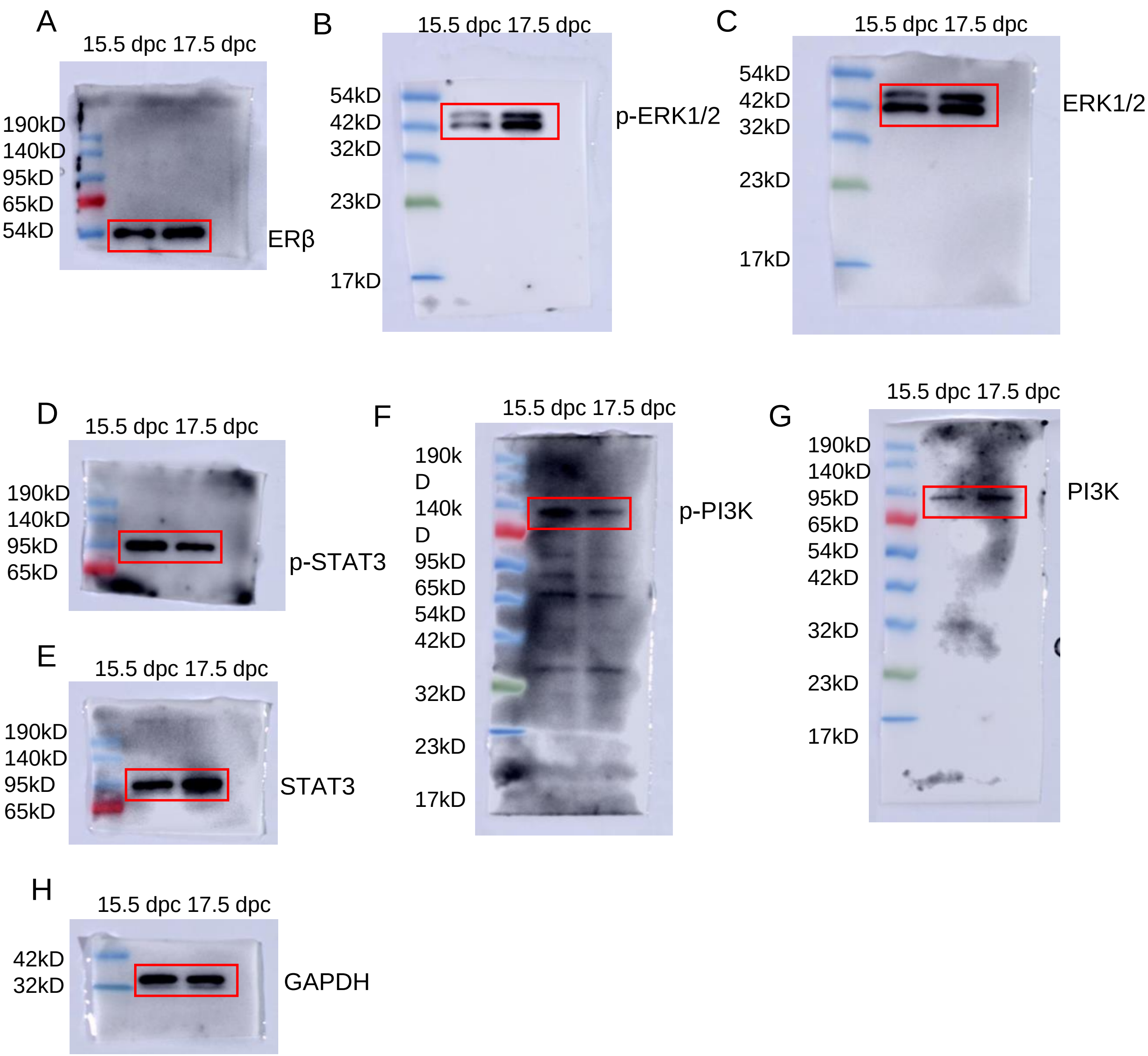


**Original images 2:** western blot. A: ERβ B: p-ERK1/2 C: ERK1/2 D: p-STAT3 E: STAT3 F: p-PI3K G: PI3K H: GAPDH

ERβ, p-ERK1/2, p-STAT3 and p-PI3K were the target proteins, GAPDH, ERK1/2, STAT3 and PI3K were the internal reference protein. In the original figure of immunoblot for all proteins, the first column is 15.5 dpc embryonic mouse ovaries and the second column is 17.5 dpc embryonic mouse ovaries.
